# Self-Collected Samples to Detect SARS-CoV-2: Direct Comparison of Saliva, Tongue Swab, Nasal Swab, Chewed Cotton Pads and Gargle Lavage

**DOI:** 10.3390/jcm10245751

**Published:** 2021-12-08

**Authors:** Niko Kohmer, Lisa Eckermann, Boris Böddinghaus, Udo Götsch, Annemarie Berger, Eva Herrmann, Marhild Kortenbusch, Peter Tinnemann, Rene Gottschalk, Sebastian Hoehl, Sandra Ciesek

**Affiliations:** 1 Institute for Medical Virology, University Hospital, Goethe University Frankfurt, 60596 Frankfurt, Germany; niko.kohmer@kgu.de (N.K.); lisa.eckermann@web.de (L.E.); annemarie.berger@kgu.de (A.B.); marhild.kortenbusch@kgu.de (M.K.); rene.gottschalk@stadt-frankfurt.de (R.G.); sandra.ciesek@kgu.de (S.C.); 2 Health Protection Authority, City of Frankfurt, 60313 Frankfurt, Germany; boris.boeddinghaus@stadt-frankfurt.de (B.B.); udo.goetsch@kgu.de (U.G.); peter.tinnemann@stadt-frankfurt.de (P.T.); 3 Institute of Biostatistics and Mathematical Modelling, Goethe University Frankfurt, 60596 Frankfurt, Germany; herrmann@med.uni-frankfurt.de; 4 German Centre for Infection Research, External Partner Site, 60323 Frankfurt, Germany; 5 Fraunhofer Institute for Molecular Biology and Applied Ecology (IME), Branch Translational Medicine and Pharmacology, 60596 Frankfurt, Germany

**Keywords:** SARS-CoV-2, self-collected samples, PCR, COVID-19, saliva, gargle lavage, nasal swab

## Abstract

Testing for Severe Acute Respiratory Syndrome Coronavirus 2 (SARS-CoV-2) by RT-PCR is a vital public health tool in the pandemic. Self-collected samples are increasingly used as an alternative to nasopharyngeal swabs. Several studies suggested that they are sufficiently sensitive to be a useful alternative. However, there are limited data directly comparing several different types of self-collected materials to determine which material is preferable. A total of 102 predominantly symptomatic adults with a confirmed SARS-CoV-2 infection self-collected native saliva, a tongue swab, a mid-turbinate nasal swab, saliva obtained by chewing a cotton pad and gargle lavage, within 48 h of initial diagnosis. Sample collection was unsupervised. Both native saliva and gargling with tap water had high diagnostic sensitivity of 92.8% and 89.1%, respectively. Nasal swabs had a sensitivity of 85.1%, which was not significantly inferior to saliva (*p* = 0.092), but 16.6% of participants reported they had difficult in self-collection of this sample. A tongue swab and saliva obtained by chewing a cotton pad had a significantly lower sensitivity of 74.2% and 70.2%, respectively. Diagnostic sensitivity was not related to the presence of clinical symptoms or to age. When comparing self-collected specimens from different material, saliva, gargle lavage or mid-turbinate nasal swabs may be considered for most symptomatic patients. However, complementary experiments are required to verify that differences in performance observed among the five sampling modes were not attributed to collection impairment.

## 1. Introduction

Testing for SARS Coronavirus 2 (SARS-CoV-2) by reverse transcription polymerase chain reaction (RT-PCR) from a respiratory specimen collected by healthcare professionals is considered the gold standard [1]. However, another molecular based method, droplet digital PCR, is gaining attention as a novel highly sensitive and reliable PCR technique [2,3]. Frequent testing for SARS-CoV-2, especially in vulnerable settings, such as long-term care facilities and hospitals, can help to limit outbreaks and prevent severe illness [4,5]. Nasopharyngeal swabs are commonly used. However, these samples may be perceived as invasive and painful. This, in turn, could interfere with adherence to testing strategies, especially in the case of repeated testing in children, but also in adults. Nasopharyngeal swab collection also has other disadvantages: these include the fact that samples must be collected by healthcare professionals who must wear personal protective equipment. Self-collected, less invasive specimens, such as saliva or nasal swabs, may be a reasonable alternative. Several studies have investigated these alternative materials which have demonstrated considerable diagnostic sensitivity [6,7,8,9,10,11,12,13,14,15,16]. But few of these studies have directly compared several different self-collected materials, and investigated whether sample collection is difficult for the patient. Therefore, it is not possible to say which self-collected material should be preferred over other options, especially when sample collection is unsupervised, and therefore subject to improper collection techniques. It is also not clarified whether the presence of certain symptoms should determine which material should be tested. For example, it has not been determined whether the use of a nasal swab in the presence of rhinitis may increase the testing of a nasal swab. In this study, we directly compared the diagnostic sensitivity of saliva, a tongue swab, a nasal swab, chewed cotton pads, and gargle lavage with tap water in a large group of patients with known infection with SARS-CoV-2 in an unsupervised sample collection. As secondary objectives, we also set out to determine whether certain materials were more appropriate in certain age groups or in the presence of certain symptoms. In addition, we investigated whether the duration since the last meal or tooth brushing had an influence on test sensitivity, and also surveyed the study participants whether they experienced difficulty in collecting the samples.

## 2. Materials and Methods

### 2.1. Recruitment of Study Participants

Individuals who were tested for SARS-CoV-2 at the Health Department of the City of Frankfurt (Germany) or at the test center of the Kassenärztliche Vereinigung Frankfurt Messe (Germany) were informed about the aims of the study and invited to participate. Written informed consent was obtained from all the study participants. Samples were collected from November 2020 to April 2021. Inclusion in the study was only applicable in the case of a positive RT-PCR in the professionally collected sample. Testing for SARS-CoV-2 was indicated due to recent exposure to a person with COVID-19 or for other reasons, such as the presence of symptoms of COVID-19. Patients 18 years of age or older were eligible to participate.

### 2.2. Collection of Samples and Questionnaire

Patients who received a positive result in the original nasopharyngeal swab and who had consented to participate in the study received instructions and material to perform five different self-collected samples. The collection of the samples was not supervised. Samples collected within 48 h after transmission of the original test result were analysed in the study. The order in which the samples were collected was standardized. The tests were (1) mid-turbinate nasal swab with using one dry swab for collection. The swab was inserted through the nostril straight back one inch and rotated on both sides. Study participants were not instructed to blow their nose prior to sample collection. (2) Tongue swab. The posterior part of the tongue was to be swabbed repeatedly in a turning motion with a dry swab. (3) Saliva. At least 0.5 mL native saliva were to be provided in a sterile container. (4) Cotton swab with saliva. A dry cotton roll was to be placed between the molars and chewed down on four to five times and each side to collect saliva. (5) Gargle lavage. 10mL of tap water were gargled for 15 s in the throat, and the solution was spit into a sterile container. Self-collected materials were transported at room temperature, and further processed on the same day, usually within 12 h after sample collection.

In a questionnaire, sex, age, symptoms present at the time of testing and subjective severity (mild/moderate/severe), duration since onset of symptoms, difficulty in sampling, time since last meal, and time since brushing teeth were recorded.

A telephone hotline could be called by study participants if they had questions about the study or needed assistance, such as difficulty with sample collection.

### 2.3. RT-PCR Analysis of Self-Collected Specimens

All self-collected specimens were tested at the Institute of Medical Virology, Goethe University Frankfurt. Dry swabs (nasal swab, tongue swab) were suspended in 2 mL of phosphate-buffered saline (PBS) and incubated for 5 min prior to further processing. Depending on the viscosity in the self-collected specimen and to achieve the required input volume, saliva was diluted up to 1:2.5. The chewed cotton pads with saliva were suspended in 4 mL of PBS, compressed, and incubated for 5 min. Gargle solution was used native, without further dilution. Samples were stored at −80 °C until nucleic acid extraction and PCR-based testing was performed. Specimens were extracted using the QIAsymphony and the DSP virus/pathogen midi kit (both Qiagen GmbH, Hilden, Germany) according to the manufacturers’ protocols. Realtime RT-PCR (rRT-PCR) analysis was performed using the RealStar^®^ SARS-CoV-2 RT-PCR Kit 1.0 (Altona Diagnostics GmbH, Hamburg, Germany) [17] on the ABI PRISM^®^ 7500 Analyser (Applied Biosystems, Waltham, MA, USA) according to the manufacturers´ specifications. Three quantitative comparison samples containing 10^5^, 10^6^, and 10^7^ SARS-CoV-2 (BetaCoV/Munich/ChVir984/2020) RNA copies/mL were used to generate a standard curve and to calculate the viral RNA copies/mL. Equivocal test results were excluded from the analysis of testing sensitivity.

### 2.4. Mutation Screening of Self-Collected Specimens

To identify potential SARS-CoV-2 variants of concern (VOCs), self-collected specimens yielding the highest concentration of SARS-CoV-2 RNA of each individual were further screened for the presence of the N501Y, del69/70, and E484K spike mutations using the Allplex™ SARS-CoV-2 Variants I Assay (Seegene Inc., Seoul, South Korea) [18] on the CFX96 Touch Real-Time PCR Detection System (Bio-Rad Laboratories Inc., Hercules, CA, USA). The assay was used according to the manufacturer’s protocol. Initial RNA extraction was performed using the chemagic™ Viral DNA/RNA 300 Kit H96 (PerkinElmer, Waltham, MA, USA) on a Thermo Scientific™ KingFisher™ Flex Purification System (Thermo Fisher Scientific GmbH, Dreieich, Germany), according to the manufacturer’s protocol. Samples harbouring the N501Y and the Δ69/70 mutation were defined as Alpha variant. All other samples were defined as non-Alpha.

### 2.5. Statistical Analysis

Fleiss’ kappa index (K value), Pearson correlation, contingency coefficient analysis (Chi square) and McNemar’s test were performed using IBM SPSS Statistics 27 (IBM Corp., Armonk, NY, USA). K value interpretations were categorized as follows: <0.20 was poor, 0.21–0.40 was fair, 0.41–0.60 was moderate agreement, 0.61–0.80 was substantial agreement, and 0.81–1.00 was almost perfect agreement [19]. Correlation and contingency coefficients were interpreted according to Pearson. Confidence intervals for sensitivity were calculated using VassarStats 24 [20]. The mixed-effects model, Turkey’s multiple comparison test and two-tailed *t*-tests (α = 0.05) were performed using GraphPad Prism 9 (GraphPad Software, San Diego, CA, USA).

## 3. Results

### 3.1. Characterization of the Study Participants and Symptomology

Patients were assigned to age groups. Out of 101 study participants who reported their age, 23 were 18 to 30 years, 30 were 31 to 40 years, 25 were 41 to 50 years of age, 16 were 51 to 60 years of age, and 6 were 61 to 70 years of age. One was older than 70 years. One study participant did not report his age.

The symptoms reported by the study participants are listed in Table 1 and Table S1. Both rhinitis and cough were occurred very frequently, and were reported by 79 of the 101 participants (78.2%). One participant reported having no symptoms.

### 3.2. Specimen Sensitivity and Virus Concentration

All five self-collected specimens were provided by 102 individuals, of whom 53 were males and 49 were females. The results of the RT-PCR tests, and the resulting sensitivities are shown in Table 2 and Table S2. The cycle threshold (Ct) values for the E gene ranged between 13.73 and 39.48, corresponding to a viral load of 8.97 to 1.27 log_10_ RNA copies/mL. Saliva had a sensitivity of 92.8%, gargle solution of 89.1%, mid-turbinate nasal swabs of 85.1%, tongue swab of 74.2%, and chewed cotton pads of 70.1%. Diagnostic sensitivity of mid-turbinate nasal swabs was not significantly inferior to saliva (*p* > 0.05) in McNemar’s test. Tongue swabs and saliva collected by chewing a cotton pad had a significantly lower sensitivity than the nasal swab and saliva (*p* ≤ 0.031) (Table S3).

We also examined the sensitivity for selected samples with a viral load of ≥6 log_10_ RNA copies/mL in the original nasopharyngeal swab. Saliva had the highest sensitivity of 94.1% in these samples, followed by the nasal swabs and gargle lavage (both 88.2%), tongue swab (76.5%) and chewed cotton pads (70.6%).

The Fleiss’ kappa coefficient for the qualitative rRT-PCR results of the five self-collected specimens was 0.445 (standard error = 0.034, 95% CI = 0.378–0.512), demonstrating a moderate agreement between the different specimens.

**Figure 1:**
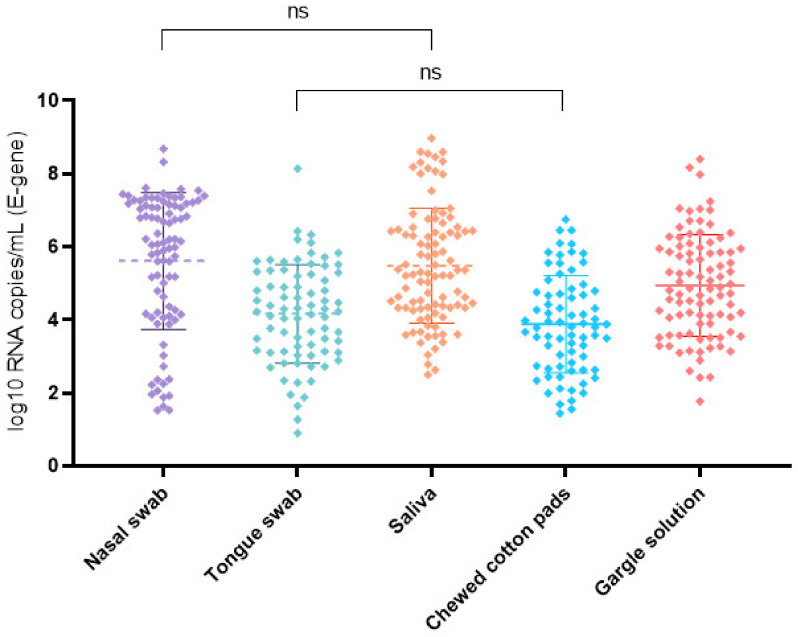
SARS-CoV-2 log_10_ RNA copies/mL for the E gene and examined self-collected specimens in order of collection including mean and standard deviation bars. The nasal swab and saliva had the highest mean viral load. The viral load was significantly lower (*p* < 0.05) in all other specimens. ns = no significant difference in the virus concentrations between the analyzed specimens.

The log_10_ SARS-CoV-2 RNA copies/mL for the E gene and different self-collected specimens are shown in Figure 1. The nasal swab had the highest mean SARS-CoV-2 RNA concentration with 5.61 (5.22–6.01 95% CI; *n* = 88). Analysis using a mixed-effects model revealed a significant difference (*p* < 0.0001) in virus concentrations between the self-collected specimens. In the Turkey’s multiple comparison tests, the virus concentration in the saliva samples was not significantly lower, with 5.48 (5.16–5.80 95% CI; *n* = 96). All other samples had a significantly lower viral concentration (Table S4).

### 3.3. Prevalence of the SARS-CoV-2 Variant Alpha and Mean Virus Concentration

To analyze the prevalence of variant of concern (VOC) Alpha, specimens were screened for the presence of SARS-CoV-2 spike mutations N501Y and Δ69/70. 55.5% (55/99) of the individuals were infected with SARS-CoV-2 harboring the N501Y and Δ69/70 mutations, indicating the presence of VOC Alpha. 12.1% (12/99) of samples had the Δ69/70 mutation only, 1% (1/99) had the N501Y mutation only. These samples were defined as non-Alpha. In 3% (3/102) of the cases, the assay was unable to provide a valid result.

The mean SARS-CoV-2 log_10_ RNA copies/mL for the Alpha and non-Alpha variants in self-collected specimens are shown in Table 3. In the samples with key mutations of the alpha variant, the virus concentration was significantly higher in nasal swab, native saliva, and gargle lavage (*p* < 0.05). The virus concentration was also higher in all other samples with the Alpha variant, but did not reach statistical significance (Table 3).

Specimens containing the Alpha variant had a higher sensitivity than the non-Alpha variant specimens (Table S5). However, differences in sensitivity were only significant for chewed cotton pads, and nasal and tongue swabs in the Fisher’s exact test (*p* < 0.05) with an Odds ratio of 4.4 (1.7–11.6 95% CI), 9.1 (1.9–43.8 95% CI) and 3.4 (1.2–9.3 95%CI), respectively.

### 3.4. Correlation of Symptoms and Testing Sensitivity of the Self-Collected Samples

Next, we examined the association between the presence of the symptoms and the sensitivity of the self-collected specimens. Using the Pearson Chi Squared test and by considering symptom severity, no significant association could be observed for rhinitis, cough, sore throat, fever, and gastrointestinal symptoms (Table S6). For the symptoms of loss of smell and taste, there was significant negative associations with testing sensitivity in the nasal swab and in saliva (Fisher’s exact test *p* = 0.001 and *p* = 0.039; Odds Ratio (OR) of 0.0126 (0.0286–0.3673 95% CI) and 0.1392 (0.0249–0.7777 95%CI)), indicating that, in the presence of this symptom, the nasal swab and saliva were inferior materials to use (Table S7). In addition, there was no significant association between a specific age group and testing sensitivity (Table S8).

### 3.5. Symptom Onset and Virus Concentration

The duration between the onset of symptoms and the time-point of self-sampling ranged from 1 to 28 days (mean 5.1 days). In the Pearson correlation analysis, there was a significant correlation (*p* < 0.05) between symptom onset and virus concentration in all self-collected materials. A moderate negative linear relationship was observed for the gargle solution, and a weak negative linear relationship for the chewed cotton pads, nasal swab, saliva, and tongue swab (Table S9). On average, samples with the Alpha variant were collected more than one day later than non-Alpha samples (range 1.19 to 1.89 days for the average value of the five different samples). We also examined whether there was a significant linear relationship between the hours since the last meal, as well as the time since the last brushing of teeth and the viral concentration (Tables S10 and S11). No such relationship could be determined in the self-collected materials. The virus concentrations in tongue swab demonstrated the highest correlation with the time since last brushing teeth: a weak positive linear relationship (r = 0.226); however, the correlation was not statistically significant (*p* = 0.051).

### 3.6. Subjective Difficulty in Collecting the Samples

Using the questionnaire, we investigated the subjective difficulties in collecting the samples (Table S12). The majority of study participants reported no difficulty with any of the specimens. The nasal swab had the highest percentage of participants reporting difficulty (16.6%).

## 4. Discussion

The use of self-collected samples, without supervision by an expert, can be helpful in testing strategies. Since they are a more convenient way to test, they can also help improve adherence. This is especially important for repeated testing in hazardous environments. In our direct head-to-head comparison, both native saliva and gargling with tap water had high diagnostic sensitivity in the unsupervised sample collection process. Saliva sampling correctly detected SARS-CoV-2 infection in 92.8% of study participants. Gargling with plain tap water identified 89.1%. Previous studies have determined that native saliva is a useful material to test for SARS-CoV-2 by RT-PCR, with fair or good agreement to the nasopharyngeal swab in symptomatic and asymptomatic patient [9,10,11]. However, other studies found only moderate concordance [13]. The presence of symptoms may increase the suitability of saliva samples [21]. In our study, almost all participants were symptomatic. Favorable data also support the use of gargling or throat wash with saline, reporting high sensitivity compared with a nasopharyngeal swab [8,12,15,22]. In our study, we achieved high sensitivity in the RT-PCR assay using plain tap water. Both saliva and gargling water can be collected at very low costs, and require the availability of a sterile container.

The mid-turbinate nasal swab had lower sensitivity of 85.1%, but this difference in detection rate from native saliva and gargling water was not statistically significant. Native saliva, gargling lavage, and the nasal swab also had the highest sensitivity in samples with high viral loads. The presence of nasal symptoms did not increase the number of study participants detected, indicating that symptom-guided use of certain materials did not increase the detection rate. Nasal swabs, both from the anterior and mid-turbinate nose, have previously been demonstrated to be a viable material, but with lower sensitivity compared with nasopharyngeal swabs [6,7,23]. Nasal swabs had the highest rate of participants who found sample collection difficult (16.6%) (Supplementary Table S12).

Chewed cotton pads as an alternative way to collect saliva, and tongue swabs both were significantly inferior in the rate of detection, with a sensitivity of 74.2% and 70.2%, respectively. These samples, as therefore expected, also had a lower mean viral load when compared with saliva, gargle lavage, and mid-turbinate nasal swabs (Figure 1). Data on tongue swabs are rare. One study determined an estimated sensitivity of 89% when compared to the nasopharyngeal swab, which was only faintly lower than nasal and mid-turbinate samples [14]. There are few investigations that examined the suitability of chewed cotton pads, possibly due to an inconvenient workflow in the laboratory. One study demonstrated a significant agreement between nasopharyngeal swabs and saliva collected in a cotton pad device [16], and in other studies, rolled cotton achieved higher performance in the molecular diagnosis of an infection with SARS-CoV-2 [24,25,26,27]. Using a cotton pad to collect saliva was inferior to the collection of native saliva in a sterile container in our study. Possible explanations include insufficient sample collections (it should be noted that, in our study, dry cotton pads were collected by chewing on the samples up to ten times, while in other studies [16] the pad was chewed for a minute or more), or other pre-analytic or laboratory workflow-derived factors, leading to a better detectability of SARS-CoV-2 in native saliva in our study.

Supplying a sufficient amount of saliva in a container or gargling may be challenging for some patients, e.g., with neurological impairments or orofacial dyskinesia. In these patients, the use of a cotton pad may be an acceptable alternative to collect a saliva sample. Based on the results of our study, saliva, gargling lavage, and mid-turbinate nasal swabs can be preferentially recommended as suitable self-collected materials for testing for SARS-CoV-2 by RT-PCR. Due to local laboratory conditions, preanalytical processing of saliva or gargle water for PCR testing may be more labor intensive and time consuming, as compared to routine testing of swabs. This may also affect the suitability of the materials for certain applications. Interestingly, sensitivities were comparable across age groups 18 years and older. There was no significant association between any particular age group and qualitative rRT-PCR results.

We also investigated whether the time of the last meal before sample collection or the time when a study participant last brushed their teeth affected viral load. No significant relationship was found for any of the materials studied. However, the time passed since the last meal was less than three hours in only three patients, and therefore the significance of this finding is limited.

We examined whether the presence of the SARS-CoV-2 variant Alpha exhibited altered detection rates and viral loads compared to non-Alpha samples. Samples with the Alpha variant had higher diagnostic sensitivity, indicating that the emergence of this variant of concern did not interfere with the suitability of the self-collected samples. Higher diagnostic sensitivity is likely attributable to higher virus concentrations observed in patients infected with the Alpha variant, also observed elsewhere [28,29]. The low number of samples limits the conclusions that can be drawn from variant-specific viral loads. Since the study was conducted, the VOC Delta became the dominant strain in central Europe. The influence of this variant has not yet been investigated in our study.

There are other limitations as well. First and foremost, the samples were not collected at the same time as the original nasopharyngeal swab. This time delay of up to 48 h probably reduced the overall sensitivity of all the materials we examined in this study. The negative correlation of time since symptom onset was also observed in our study. Due to this delay in testing, the distinct suitability of self-collected samples we determined in our study may not be transferrable to earlier stages of an infection with SARS-CoV-2. The viral load of the original nasopharyngeal swab was not available for all patients, and could not be directly compared to the other samples. Moreover, since we included only study participants with confirmed infection according to the gold standard, we could not determine the extent to which these materials might be superior to the nasopharyngeal swab. The specific protocol used to collect the samples and the order in which the samples were collected was standardized in this study, but varies between different studies. Therefore, the comparability of our results with the literature is limited. In addition, we used tap water for gargle collection. The local composition and quality of tap water may affect its suitability for testing for SARS-CoV-2.

Sample collection was not supervised by healthcare professionals. This means that the results of the study reflect a situation in which sampling may have been performed inadequately, which may have biased the results. The use of instructional videos may improve the quality of sample collection, and a control if sampling was performed adequately (e.g., Human RNase P) should be considered in future studies. Through the examination of samples that were collected without supervision, we could avoid other potential biases, such as the Hawthorne effect.

We chose a standardized order of tests to minimize the influence of a prior test on the next. However, the standardized order may still have influenced testing, and therefore the results of the study.

In summary, we observed that in a direct head-to-head comparison, multiple self-collected samples offer high diagnostic sensitivity and can be recommended for most symptomatic adult subjects. When selecting a sample for unsupervised collection, native saliva, gargling water, or mid-turbinate nasal swabs should be preferred over tongue swabs or saliva collected in a chewed cotton pad in most cases. As we cannot rule out sample collection impairment among the participants or an influence due to the order of collection, complementary experiments are required to verify our findings.

## Figures and Tables

**Table 1:** Symptoms reported by the 101 study participants, all of whom had recently been confirmed to be infected with SARS-CoV-2. One participant did not provide data on symptoms.

Symptom	Present in Number of Patients, Out of 101	%
Rhinitis	79	78.2
Cough	79	78.2
Sore throat	68	67.3
Fever	50	59.5
Gastrointestinal symptoms	41	40.6
Loss of smell or taste	14	13.9
Not reporting symptoms	1	0.9

**Table 2:** Sensitivity of SARS-CoV-2 rRT-PCR for the examined self-collected specimens when compared to the initially rRT-PCR positive tested nasopharyngeal swabs.

		Chewed Cotton Pads	Nasal Swab	Saliva	Tongue Swab	Gargle Solution
Sensitivity %	all samples (*n* = 102)	70.1% (68/97) (59.8–78.8% 95% CI)	85.1% (86/101) (76.4–91.2% 95% CI)	92.8% (90/97) (85.2–96.8% 95% CI)	74.2% (72/97) (64.2–82.3% 95% CI)	89.1% (90/101) (81.0–94.2% 95% CI)
	selected nasopharyngeal swabs containing ≥6 log_10_ RNA copies/mL (*n* = 17) *	70.6% (12/17) (44–88.6% 95% CI)	88.2% (15/17) (62.3–97.9% 95% CI)	94.1% (16/17) (69.2–99.7% 95% CI)	76.5% (13/17) (49.8–92.2% 95% CI)	88.2% (15/17) (62.3–97.9% 95% CI)

Interpretations according to the manufacturers’ specifications, also considering the S-gene tested in parallel. Equivocal test results were excluded: Chewed cotton pads (*n* = 5), saliva (*n* = 5l), nasal swabs (*n* = 1), tongue swabs (*n* = 5l), and gargle solution (*n* = 1); * Ct value (E gene) ≤25.44; including one sample with a corresponding Ct value (N/RdRP gene) <22.09.

**Table 3:** Mean SARS-CoV-2 concentrations in self-collected specimens, by variant (Alpha, non-Alpha). Values depicted in mean SARS-CoV-2 log_10_ RNA copies/mL (E gene).

		Alpha Variant	Non-Alpha Variant	*p*-Value *
(a)	Chewed cotton pads	3.97 (3.57–4.36 95% CI) (*n* = 46)	3.7 (3.14–4.25 95% CI) (*n* = 25)	0.423
(b)	Nasal swab	6.35 (6.00–6.71 95% CI) (*n* = 52)	4.84 (4.11–5.58 95% CI) (*n* = 30)	**<0.0001**
(c)	Saliva	5.79 (5.38–6.21 95% CI) (*n* = 53)	5.12 (4.6–5.65 95% CI) (*n* = 40)	**0.0443**
(d)	Tongue swab	4.24 (3.90–4.58) (*n* = 48)	4.01 (3.38–4.64 95% CI) (*n* = 28)	0.474
(e)	Gargle lavage	5.36 (4.98–5.74 95% CI) (*n* = 51)	4.4 (3.97–4.83 95% CI) (*n* = 37)	**0.00012**

* unpaired two-tailed *t*-test.

## Data Availability

Data is contained within the Supplementary Materials.

## Supplementary Materials

The following are available online at https://www.mdpi.com/article/10.3390/jcm10245751/s1, Table S1: Frequency of reported symptoms and degree of severity, Table S2: SARS-CoV-2 log_10_ RNA copies/mL (E gene) in self-collected specimens, mutation screening, age, and time parameters, Table S3: Comparison of sensitivities using McNemar’s test, Table S4: Turkey’s multiple comparisons test and adjusted *p*-value of self-collected specimens, Table S5: Sensitivity of self-collected specimens regarding the Alpha and non-Alpha variant, Table S6: Association between qualitative rRT-PCR results of self-collected specimens and reported symptoms (also considering symptom severity), Table S7: Odds Ratios of reported symptoms loss of smell and taste and qualitative rRT-PCR results of nasal swab and saliva, Table S8: Age group specific sensitivity of self-collected specimens, Table S9: Pearson correlation analysis between symptom onset and SARS-CoV-2 log_10_ RNA copies/mL in self-collected materials, Table S10: Pearson correlation analysis between hours since last meal and SARS-CoV-2 log_10_ RNA copies/mL in self-collected materials, Table S11: Pearson correlation analysis between the time since last brushing teeth and SARS-CoV-2 log_10_ RNA copies/mL in self-collected materials, Table S12: Subjective difficulty in collecting the samples.
