# Supplementary material for: Self-Collected Samples to Detect SARS-CoV-2: Direct Comparison of Saliva, Tongue Swab, Nasal Swab, Chewed Cotton Pads and Gargle Lavage
Source: J Clin Med. 2021 Dec 8;10(24):5751. doi: 10.3390/jcm10245751 (PMC8709431; doi:10.3390/jcm10245751)
Supplement: Supplementary file 1 [file jcm-10-05751-s001.zip › Supplemental Material.pdf]

Supplemental material to

Self-collected samples to detect SARS-CoV-2: Direct comparison of saliva, tongue swab, nasal swab, chewed cotton pads and gargle lavage

Contents

Supplemental Table S1 2
Supplemental Table S2 3
Supplemental Table S3 7
Supplemental Table S4 8
Supplemental Table S5 9
Supplemental Table S6 10
Supplemental Table S7 11
Supplemental Table S8 12
Supplemental Table S9 13
Supplemental Table S10 14
Supplemental Table S11 15
Supplemental Table S12 16

Table S1

Frequency of reported symptoms and degree of severity.

Symptom	Mild	Moderate	Severe
Rhinitis 78.2% (79/101)	54.4% (43/79)	43% (34/79)	2.5% (2/79)
Cough 78.2% (79/101)	59.5% (47/79)	31.6% (25/79)	8.9% (7/79)
Sore throat 67.3% (68/101)	66.2% (45/68)	23.5% (16/68)	10.3% (7/68)
Fever 59.5% (50/101)	58.0% (29/50)	30% (15/50)	12.0% (6/50)
Gastrointestinal symptoms 40.6% (41/101)	73.2% (30/41)	26.8% (11/41)	-
Loss of smell and taste 13.9% (14/101)			
No symptoms present 1.0% (1/101)			

Table S2

SARS-CoV-2 log_{10} RNA copies/mL (E gene) in self-collected specimens, mutation screening, age and time parameters.

Days since symptom onset	Nasal swab	Tongue swab	Saliva	Chewed cotton pads	Gargle solution	Mutation Screening (SARS-CoV-2 Spike Mutation N501Y; Δ69/70; E484K)	Distance from last meal (hours)	Distance from brushing teeth (hours)	Age (years)
	log10 RNA copies/mL (E gene)								
6	-	1.95	2.78	1.99	4.13	Δ69/70	1.0	5.0	31-40
3	2.22	-	2.50	-	4.11	-	13.0	7.0	18-30
7	4.15	5.32	5.74	2.25	3.10	-	13.0	9.0	51-60
9	3.88	-	3.21	-	3.23	-	n/a	n/a	51-60
5	3.32	3.11	6.39	3.67	5.98	-	1.5	6.0	18-30
8	-	-	-	-	-	failed	10.0	10.0	41-50
6	2.06	2.34	6.53	3.00	3.46	-	2.0	2.0	18-30
5	-	-	-	-	2.42	Δ69/70	14.0	12.0	31-40
8	4.63	5.48	7.05	4.85	4.81	Δ69/70	10.0	15.0	41-50
4	2.36	2.81	5.24	3.36	3.55	-	12.0	9.0	51-60
n/a	1.53	4.89	5.50	3.78	5.18	-	12.0	9.0	18-30
4	-	1.27	3.67	-	3.89	RdRP gene	12.0	9.0	41-50
9	-	-	4.77	-	3.27	Δ69/70	0.3	10.0	18-30
28	2.25	-	-	-	-	failed	0.0	0.5	51-60
n/a	-	-	4.30	-	-	-	n/a	n/a	41-50
2	4.00	2.81	3.59	-	4.08	-	13.5	10.0	31-40
8	-	-	4.33	-	3.29	Δ69/70	12.0	8.0	41-50
8	-	-	3.84	-	3.52	-	13.0	9.0	41-50
5	4.79	-	4.18	-	3.58	N501Y	16.0	12.0	31-40
8	-	1.64	3.54	2.44	3.12	-	n/a	n/a	18-30
3	7.38	6.20	5.27	-	4.51	-	8.0	7.0	61-70
6	5.62	2.32	4.25	1.44	3.15	-	10.0	9.0	18-30
8	5.22	4.32	4.34	4.66	6.24	-	11.0	10.0	51-60
11	2.73	-	3.61	-	-	-	10.0	9.0	18-30
3	6.80	4.30	4.53	2.07	5.75	-	9.0	8.5	51-60
3	6.20	4.62	5.87	3.49	4.15	-	12.0	12.0	41-50
4	7.46	8.14	8.46	6.45	6.54	-	13.0	12.0	51-60
9	4.36	3.13	4.07	2.44	-	Δ69/70	13.0	11.0	31-40
4	1.96	3.27	5.62	3.13	4.85	-	14.0	13.0	41-50
3	7.40	5.35	6.08	4.60	6.39	N501Y; Δ69/70	15.0	9.0	18-30
4	7.27	4.80	7.53	5.78	7.98	-	13.0	7.0	61-70
6	3.89	5.51	6.30	4.18	5.32	-	13.0	11.0	18-30
2	5.01	4.53	5.36	3.30	3.67	-	14.0	0.2	41-50
7	1.88	2.28	4.41	-	-	-	16.0	13.0	41-50

8	-	2.72	4.63	-	3.28	-	8.0	8.0	18-30
5	5.99	-	4.62	2.66	4.98	-	10.0	9.5	41-50
17	-	-	4.00	-	-	-	14.0	11.0	31-40
3	7.35	4.90	4.74	2.41	4.20	N501Y; Δ69/70	10.0	9.0	51-60
5	6.36	3.49	5.21	3.30	4.89	-	10.5	11.0	31-40
5	6.77	5.61	6.81	5.55	6.38	-	11.0	6.8	51-60
7	-	-	3.37	-	2.43	-	10.0	8.0	41-50
1	8.32	4.04	4.33	3.55	3.58	N501Y; Δ69/70	14.3	11.0	31-40
2	7.58	4.30	5.31	3.53	3.51	N501Y; Δ69/70	11.5	11.0	41-50
5	6.11	-	4.33	1.79	4.06	N501Y; Δ69/70	14.0	12.0	41-50
6	4.20	-	4.33	2.74	4.86	N501Y; Δ69/70	15.0	13.0	18-30
3	7.26	3.14	6.54	4.31	6.62	N501Y; Δ69/70	12.0	9.5	51-60
3	5.17	3.03	6.44	2.34	4.42	N501Y; Δ69/70	7.0	7.0	31-40
2	6.65	5.83	8.15	4.68	5.85	Δ69/70	15.0	12.0	41-50
3	6.84	3.66	4.06	-	4.67	N501Y; Δ69/70	14.0	10.0	51-60
1	6.21	2.72	3.80	2.00	2.60	N501Y; Δ69/70	1.0	4.0	31-40
4	5.60	3.66	3.56	5.37	7.05	N501Y; Δ69/70	n/a	n/a	61-70
3	7.18	3.77	4.38	2.63	5.47	N501Y; Δ69/70	8.0	12.0	41-50
9	-	-	4.87	-	-	N501Y; Δ69/70	11.0	9.0	31-40
7	4.16	2.70	5.19	2.60	4.52	N501Y; Δ69/70	12.0	12.0	41-50
3	7.33	5.25	6.42	3.90	6.28	N501Y; Δ69/70	11.0	10.5	18-30
3	5.74	4.39	6.92	4.97	5.28	N501Y; Δ69/70	13.0	12.0	18-30
4	7.08	4.04	6.47	3.52	5.82	N501Y; Δ69/70	12.0	10.0	31-40
6	6.74	2.89	4.51	4.15	4.48	N501Y; Δ69/70	16.0	12.0	51-60
4	7.34	-	4.42	-	4.13	N501Y; E484K	14.0	10.0	51-60
6	4.10	3.77	5.03	4.27	4.44	N501Y; Δ69/70	12.0	24.0	18-30
2	7.07	6.33	8.31	6.45	8.40	N501Y; Δ69/70	15.0	12.0	31-40
n/a	4.06	-	-	-	3.51	Δ69/70	n/a	n/a	
2	8.68	4.72	6.90	5.09	5.86	Δ69/70	14.0	11.0	41-50
4	5.83	3.10	5.38	3.81	4.70	N501Y; Δ69/70	12.0	9.0	31-40
4	7.28	3.42	8.97	5.86	6.01	N501Y; Δ69/70	12.0	9.0	31-40

3	6.91	5.83	7.99	3.58	5.18	N501Y; Δ69/70	13.0	10.0	61-70
5	7.24	5.64	8.60	4.71	4.56	Δ69/70	3.0	8.0	>70
4	5.18	4.60	5.36	3.80	5.27	N501Y; Δ69/70	1.0	6.0	31-40
5	7.03	4.08	6.43	6.08	5.95	N501Y; Δ69/70	0.5	0.5	41-50
6	6.07	0.90	5.05	5.58	5.60	N501Y; Δ69/70	10.0	1.0	51-60
4	7.39	6.43	6.31	4.32	7.04	N501Y; Δ69/70	15.0	22.0	41-50
3	6.79	4.40	7.00	4.79	6.71	N501Y; Δ69/70	12.0	8.0	31-40
5	7.61	3.68	5.21	-	5.66	N501Y; Δ69/70	13.0	11.0	31-40
n/a	1.52	-	-	4.76	-	N501Y; Δ69/70	n/a	n/a	51-60
10	2.37	-	3.04	2.63	3.14	Δ69/70	9.0	15.0	18-30
7	3.02	3.02	3.39	-	5.04	N501Y; Δ69/70	7.5	7.0	31-40
3	6.68	5.72	8.55	6.75	8.17	N501Y; Δ69/70	11.0	10.0	31-40
3	7.07	5.53	5.22	3.88	4.25	N501Y; Δ69/70	1.0	1.0	41-50
2	7.54	5.59	8.07	5.56	6.12	N501Y; Δ69/70	1.0	4.0	18-30
4	5.89	3.32	6.39	-	3.62	N501Y; Δ69/70	4.0	7.0	18-30
1	7.44	4.15	5.79	2.12	4.67	N501Y; Δ69/70	3.0	17.0	31-40
n/a	1.63	-	4.62	-	4.72	N501Y; Δ69/70	13.0	12.0	31-40
3	-	-	-	-	-	failed	5.0	10.0	18-30
4	7.34	4.52	5.80	4.02	5.85	N501Y; Δ69/70	12.5	9.0	18-30
7	4.27	-	3.65	-	1.77	N501Y; Δ69/70	12.0	10.0	31-40
8	6.76	5.65	6.77	4.42	7.01	N501Y; Δ69/70	12.0	9.0	61-70
4	6.21	3.16	6.33	3.04	4.19	N501Y; Δ69/70	11.0	9.0	31-40
4	6.83	6.11	8.00	5.85	6.26	N501Y; Δ69/70	11.0	12.0	31-40
5	4.06	4.11	8.19	5.26	5.56	Δ69/70	11.0	9.5	31-40
2	5.60	1.88	4.46	3.58	7.24	N501Y; Δ69/70	9.0	9.0	31-40
5	5.79	5.08	2.63	3.96	3.89	N501Y; Δ69/70	14.0	10.0	41-50
6	1.92	-	3.60	-	-	N501Y; Δ69/70	13.0	14.0	18-30
5	7.16	4.23	6.03	3.50	5.31	N501Y; Δ69/70	8.5	8.0	51-60
5	7.21	5.20	8.34	5.82	6.09	N501Y; Δ69/70	12.0	12.0	41-50

3	7.36	5.29	5.51	3.94	5.82	N501Y; Δ69/70	10.0	9.5	31-40
2	5.96	4.91	4.49	1.69	5.07	N501Y; Δ69/70	3.0	5.0	18-30
4	7.12	4.22	6.76	6.10	6.98	N501Y; Δ69/70	4.0	6.0	18-30
7	7.19	4.47	6.27	3.98	6.71	N501Y; Δ69/70	10.0	16.0	41-50
3	7.13	4.80	6.65	4.12	5.37	N501Y; Δ69/70	12.0	24.0	31-40
2	5.18	5.37	8.60	2.83	5.95	N501Y; Δ69/70	12.0	12.0	41-50
4	6.15	3.49	6.08	1.55	2.89	N501Y; Δ69/70	1.0	6.0	31-40
4	6.05	3.52	6.27	2.72	6.35	N501Y; Δ69/70	1.0	6.0	61-70

- = negative/not detected

n/a = not available

Table S3

Comparison of sensitivities using McNemar’s test.

**Sensitivity of specimen**	**p-value**
Nasal swab vs. Gargle lavage	0,424
Nasal swab vs. Saliva	0,092
Nasal swab vs. Chewed cotton pads	**0,001**
Nasal swab vs. Tongue Swab	**0,031**
Gargle lavage vs. Chewed cotton pads	**<0,0005**
Saliva vs. Gargle lavage	0,344
Saliva vs. Chewed cotton pads	**<0,0005**
Tongue swab vs. Gargle lavage	**0,002**
Tongue swab vs. saliva	**<0,0005**
Tongue swab vs. Chewed cotton pads	0,388

Table S4

Turkey’s multiple comparisons test and adjusted p-value of self-collected specimens.

**Tukey's multiple comparisons test**	**Adjusted p value**
Chewed cotton pads vs. Nasal swab	**<0,0001**
Chewed cotton pads vs. Saliva	**<0,0001**
Chewed cotton pads vs. Tongue swab	0,3857
Chewed cotton pads vs. Gargle lavage	**<0,0001**
Nasal swab vs. Saliva	0,9586
Nasal swab vs. Tongue swab	**<0,0001**
Nasal swab vs. Gargle lavage	**0,003**
Saliva vs. Tongue swab	**<0,0001**
Saliva vs. Gargle lavage	0,0018
Tongue swab vs. Gargle lavage	**<0,0001**

Table S5

Sensitivity of self-collected specimens regarding the Alpha and non-Alpha variant.

	Sensitivity		Fisher's exact test (p-value)	Odds ratio
	Alpha variant	Non-Alpha variant		
Chewed cotton pads	**84.9%** (71.9-92.8% 95% CI) (45/53)	**56.1%** (39.9-71.2% 95% CI) (23/41)	**0.003**	4.4 (1.7-11.6 95% CI)
Nasal swab	**96.3%** (86.4-99.4% 95% CI) (53/55)	**74.4%** (58.5-86% 95% CI) (32/43)	**0.002**	9.1 (1.9-43.8 95% CI)
Saliva	**96.2%** (85.9-99.3% 95% CI) (51/53)	**95.1%** (82.2-99.2% 95% CI) (39/41)	1.000	1.3 (0.2-9.7 95% CI)
Tongue swab	**86.3%** (73.1-93.8% 95% CI) (44/51)	**65.1%** (49-78.5% 95% CI) (28/43)	**0.026**	3.4 (1.2-9.3 95%CI)
Gargle lavage	**94.4%** (83.7-98.6% 95% CI) (51/54)	**88.6%** (74.6-95.7% 95% CI) (39/44)	0.461	2.2 (0.5-9.7 95% CI)

Table S6

Association between qualitative rRT-PCR results of self-collected specimens and reported symptoms (also considering symptom severity).

	Rhinitis	Cough	Sore throat	Fever	Gastrointestinal symptoms	
Chewed cotton pads	0.413	0.386	0.122	0.073	0.447	p-value*
Nasal swab	0.812	0.129	0.465	0.187	0.471	
Saliva	0.831	0.879	0.726	0.581	0.452	
Tongue swab	0.867	0.677	0.178	0.634	0.687	
Gargle lavage	0.621	0.718	0.662	0.174	0.081	

*Pearson Chi Square test

Table S7

Odds Ratios of reported symptoms loss of smell and taste and qualitative rRT-PCR results of nasal swab and saliva.

	rRT-PCR	Loss of smell and taste		Odds Ratio
		Not present	Present	
Nasal swab	Negative	8	7	0.0126 (0.0286-0.3673 95% CI)
	Positive	78	7	9.75 (2.7229-34.9119 95% CI)
Saliva	Negative	3	3	0.1392 (0.0249-0.7777 95%CI)
	Positive	79	11	7.1818 (1.2858-40.1133 95%CI)

Table S8

Age group specific sensitivity of self-collected specimens.

	Sensitivity					p-value*
	Age group (years)					
	18-30	31-40	41-50	51-60	61-70	
Gargle lavage	87% (20/23)	89.7% (26/29)	88.0% (22/25)	88.2% (15/17)	100.0% (5/5)	0.944
Saliva	95.2% (20/21)	92.9% (26/28)	96.0% (24/25)	87.5% (14/16)	100.0% (5/5)	0.788
Chewed cotton pads	66.7% (14/21)	70.0% (21/30)	70.8% (17/24)	73.3% (11/15)	80.0% (4/5)	0.980
Tongue swab	69.6% (16/23)	77.8% (21/27)	72.0% (18/25)	73.3% (11/15)	100.0% (5/5)	0.688
Nasal swab	81.8% (18/22)	86.7% (26/30)	72.0% (19/25)	94.1% (16/17)	100.0% (5/5)	0.436

*Pearson Chi Square test

Table S9

Pearson correlation analysis between symptom onset and SARS-CoV-2 log_{10} RNA copies/mL in self-collected materials.

Examined material	Pearson correlation coefficient (r)	two-sided significance (p-value)*	Interpretation
Nasal swab	-0.30	**0.003**	weak negative linear relationship
Tongue swab	-0.48	**<0.0005**	weak negative linear relationship
Saliva	-0.31	**0.003**	weak negative linear relationship
Chewed cotton pads	-0.40	**<0.0005**	weak negative linear relationship
Gargle lavage	-0.54	**<0.0005**	moderate negative linear relationship

*<0.05 = significant correlation

Table S10

Pearson correlation analysis between hours since last meal and SARS-CoV-2 log_{10} RNA copies/mL in self-collected materials.

Examined material	Pearson correlation coefficient (r)	two-sided significance (p-value)	Interpretation
Nasal swab	-0.013	0.909	very weak negative linear relationship
Tongue swab	0.168	0.151	very weak positive linear relationship
Saliva	-0.046	0.665	very weak negative linear relationship
Chewed cotton pads	0.212	0.078	weak positive linear relationship
Gargle lavage	0.097	0.372	very weak positive linear relationship

Table S11

Pearson correlation analysis between the time since last brushing teeth and SARS-CoV-2 log_{10} RNA copies/mL in self-collected materials.

Examined material	Pearson correlation coefficient (r)	two-sided significance (p-value)	Interpretation
Nasal swab	0.024	0.827	very weak positive linear relationship
Tongue swab	0.226	0.051	weak positive linear relationship
Saliva	0.043	0.686	very weak positive linear relationship
Chewed cotton pads	0.014	0.908	very weak positive linear relationship
Gargle lavage	0.097	0.373	very weak positive linear relationship

Table S12

Subjective difficulty in collecting the samples.

Specimen	very easy	rather easy	rather difficult	very difficult
Saliva (n=97)	80.4% (78/97)	13.4% (13/97)	6.2% (6/97)	0.0% (0/97)
Chewed cotton pads (n=96)	85.4% (82/96)	6.3% (6/96)	6.3% (6/96)	2.1% (2/96)
Gargle lavage (n=95)	78.9% (75/95)	9.5% (9/95)	10.5% (10/95)	1.1% (1/95)
Nasal swab (n=96)	51.0% (49/96)	32.3% (31/96)	15.6% (15/96)	1.0% (1/96)
Tongue swab (n=96)	60.4% (58/96)	26.0% (25/96)	10.4% (10/96)	3.1% (3/96)
